# Malignant mesothelioma of the pleura with desmoplastic histology: a case series and literature review

**DOI:** 10.1186/s12885-016-2745-8

**Published:** 2016-09-06

**Authors:** Kana Hashimoto, Yusuke Okuma, Yukio Hosomi, Tsunekazu Hishima

**Affiliations:** 1 Department of Thoracic Oncology and Respiratory Medicine, Tokyo Metropolitan Cancer and Infectious diseases Center Komagome Hospital, 3-18-22 Honkomagome, Bunkyo, Tokyo 113-8677 Japan; 2 Division of Oncology, Research Center for Medical Sciences, Jikei University School of Medicine, Tokyo, Japan; 3 Department of Pathology, Tokyo Metropolitan Cancer and Infectious diseases Center Komagome Hospital, Tokyo, Japan

**Keywords:** Desmoplastic, Mesothelioma, Chemotherapy, Prognosis

## Abstract

**Background:**

Desmoplastic malignant pleural mesothelioma (DMM) is rare histological subtype of diffuse malignant pleural mesothelioma (MPM), accounting for 5–10 % of cases. It has a poor prognosis, with direct invasion of the chest wall or lungs and distant metastases. Its pathological characteristics include dense collagen fibers in a storiform pattern. Its pretreatment pathological diagnosis is difficult, with fibrous pleuritis and reactive mesothelial hyperplasia as potential differential diagnoses.

**Case presentation:**

We retrospectively reviewed the medical charts of patients with MPM from 1996 to 2012. Among 60 patients with MPM, four patients with the desmoplastic subtype were identified and their clinical characteristics, including asbestos exposure, treatment, and prognosis, were reviewed. All of the patients with DMM were men, with a median age of 69 years (range: 63–74 years). All four patients had been exposed to asbestos. The definitive diagnosis was made histologically and the International Mesothelioma Interest Group classification was advanced (III/IV: 2/3) in all four patients. Three patients were treated with chemotherapy (two with cisplatin/pemetrexed and one with cisplatin/gemcitabine) and one patient underwent surgery. The median survival time in the patients with DMM was 3.8 months (range: 0.9–11.5 months), compared with 10.5 months in patients with other subtypes of MPM in our institution.

**Conclusions:**

DMM continues to have a poor prognosis. It is important to recognize this variant and distinguish it from pleural plaques, non-specific reactive pleural fibrosis, pleurisy, and other lung diseases.

**Electronic supplementary material:**

The online version of this article (doi:10.1186/s12885-016-2745-8) contains supplementary material, which is available to authorized users.

## Background

Malignant mesothelioma is a rare cancer arising in body cavities lined by mesothelium, commonly including the pleura [1]. A recent report suggested an increasing trend of malignant mesothelioma incidence, and that it was still associated with a poor prognosis. Asbestos exposure, often as an occupational hazard, has been clearly linked to the occurrence of malignant pleural mesothelioma (MPM) [2]. MPM has a poor prognosis, with a median survival of 4–12 months due to a lack of successful curative treatments and its diagnosis at an advanced stage [3]. MPM is categorized histologically as epithelioid, sarcomatoid, or biphasic [1, 4] subtypes, which account for 55, 15, and 30 % of MPM patients, respectively.

Desmoplastic malignant mesothelioma (DMM) was first described by Kannerstein and Churg in 1980 [5], and comprises a relatively rare, specific histological subtype of sarcomatoid tumor [6]. Since its initial report, the number of cases of DMM has continued to increase sporadically, and it is thought to constitute 5–10 % of all patients with MPM. DMM is characterized by dense, collagenized tissue (>50 %) separated by atypical cells arranged in a storiform or “patternless” pattern, present in at least 50 % of the tumor specimen. Immunohistochemical (IHC) staining is useful for making a diagnosis, although the appropriate combination of antibodies needs to be selected for a comprehensive assessment. It is also crucial to distinguish DMM from benign lesions involving pleural fibrosis because of their different treatments and prognoses. However, the characteristics of this relatively rare histological subtype of mesothelioma remain unclear.

In this study, we investigated the clinical characteristics and outcomes of four patients with DMM and conducted a literature review.

## Methods

### Database and data acquisition

We retrospectively investigated patients with a histological or cytological diagnosis of MPM at the Tokyo Metropolitan Cancer and Infectious diseases Center Komagome Hospital (Tokyo, Japan) between 1996 and 2012. We used the International Classification of Diseases (9th edition) codes to identify relevant patients from the database. The patients’ clinical data were acquired from electronic charts.

The pathological diagnoses were reviewed by a pathologist (TH) in accordance with the 2004 World Health Organization classification [7], based on hematoxylin–eosin staining and additional IHC staining. The relevant clinical features and treatment-related data for 60 patients diagnosed with MPM were retrospectively reviewed. Among these patients, four were diagnosed with DMM. Staging and best objective responses were based on the International Mesothelioma Interest Group (iMig) recommendations [8, 9]. The patients’ baseline characteristics were summarized using descriptive statistics. The results are summarized in Table 1 Also, Radiographic and pathological images of each patient are attached in the Additional files (Additional file: 1, 2, 3, 4).

**Table 1:** Patient characteristics and survival

	Gender	Asbestos exposure	Diagnostic methods	Age	Stages (IMIG Classification)	Treatment	Survival (months)
Case 1	Male	+	VATS	69	T4N3M0 Stage IV	HITC	11.6
Case 2	Male	+	VATS	73	T3N0M0 Stage III	CDDP + GEM	8.1
Case 3	Male	Unknown	VATS	74	T1N2M0 Stage III	CDDP + PEM	4.7
Case 4	Male	+	VATS	63	T4N2M0 Stage IV	CDDP + GEM	0.9

*VATS* video-assisted thoracic surgery, *HITC* hyperthermic intrathoracic chemotherapy, *CDDP* cisplatin, *GEM* gemcitabine, *PEM* pemetrexed

## Case presentations

### Case 1

A 69-year-old man was referred to the Tokyo Metropolitan Cancer and Infectious diseases Center Komagome Hospital, with complaints of cough with hemoptysis, weight loss, and an abnormal chest radiograph. He also had diabetes, dyslipidemia, and a history of tuberculosis. He had been diagnosed with non-specific pleural thickness by video-assisted thoracoscopic surgery (VATS) at a different institution 3 years previously. Chest computed tomography (CT) revealed pleural effusion with thickened pleura. VATS of the pleura was performed, and the histopathological examination revealed thickening with collagen fibrous hyperplasia and invasion of inflammatory cells, mainly comprising plasma cells. The collagen fibers were irregular, with poor alignment. These characteristics were consistent with DMM. He was also diagnosed with T4N0M0 stage IV according to iMig stage. The patient was initially treated with hyperthermic intrathoracic chemotherapy at a dose of 10 Gy in 10 fractions, with palliative intent. Although his tumor shrank by 13 %, he died suddenly of cardiopulmonary arrest at home at 11.6 months after starting the treatment.

### Case 2

A 73-year-old man was initially referred to our institution with dyspnea on exertion. No lesion was present on CT for the first 5 months, but a new lesion appeared at 5.9 months. Based on the results of VATS, he was diagnosed with DMM of T3N0M0 stage III. Cells with spindle-shaped or round nuclei were loosely scattered among dense hyaline collagen fibers in the visceral pleura. IHC revealed tumor cells positive for CAM 5.2, calretinin, CD5/6, and D2-40, but negative for carcinoembryonic antigen, HBME, and thrombomodulin. He was thus diagnosed with DMM. He was treated with one cycle of cisplatin and gemcitabine combination chemotherapy, followed by carboplatin and gemcitabine because of poor performance status. However, he died of disease progression 8.1 months after initial diagnosis.

### Case 3

**Fig. 1:**
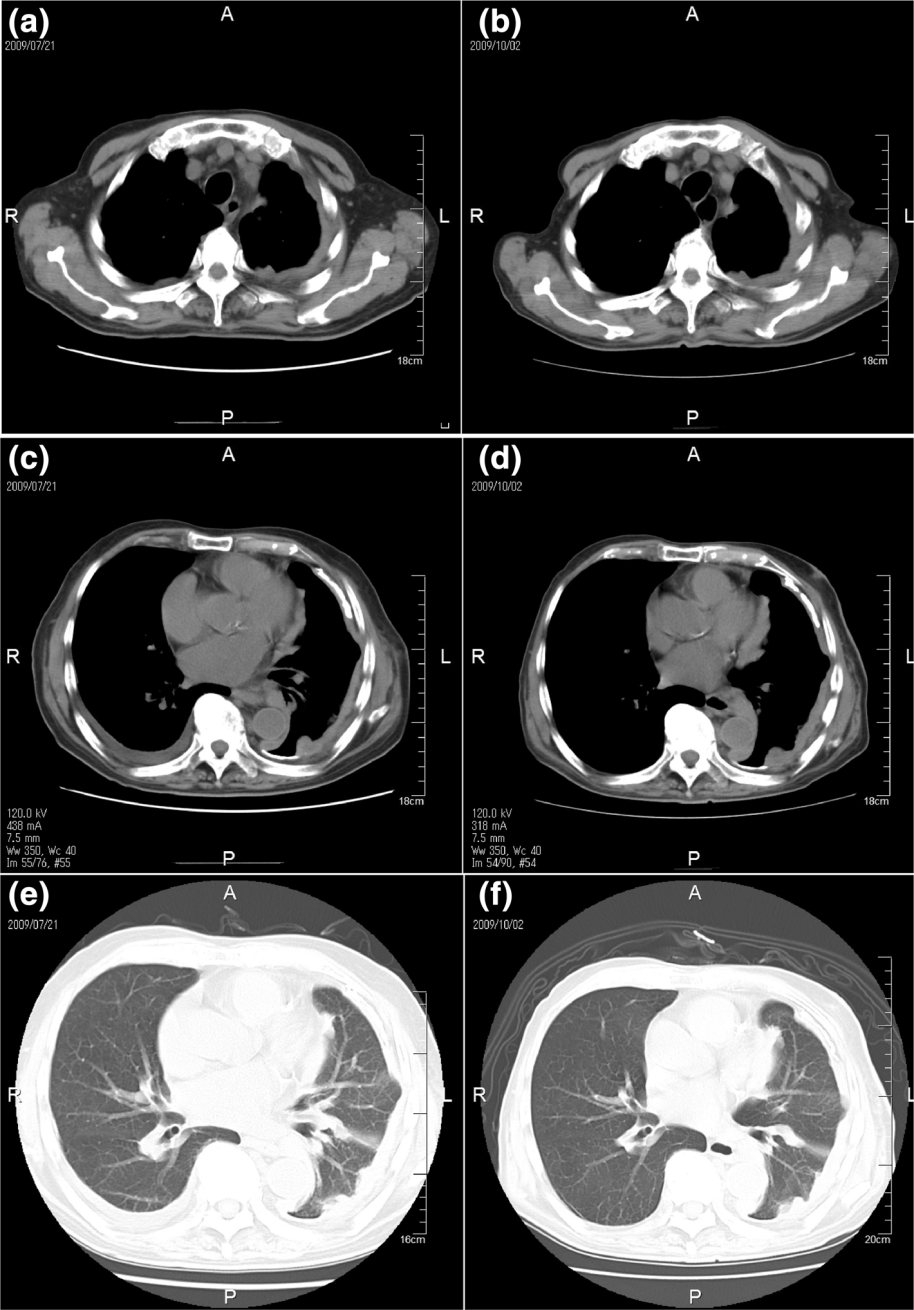
Chest computed tomography of DMM lesions (Case 3) before (**a**), (**c**), (**e**) and after cisplatin and pemetrexed chemotherapy (**b**), (**d**), (**f**)

**Fig. 2:**
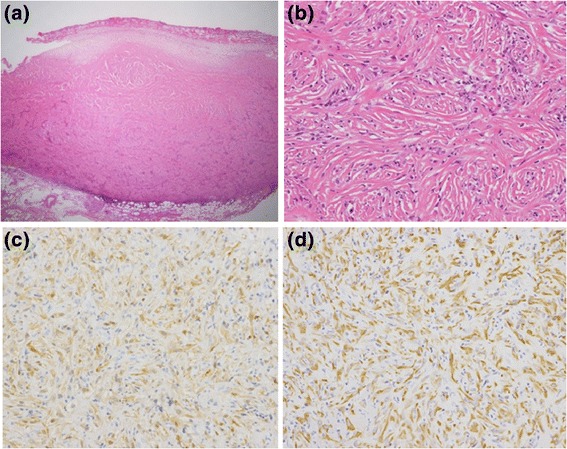
Pathological findings of a VATS-resected specimen (Case 3). Low-power image shows focal proliferation of cuboidal atypical cells with round nuclei and prominent nucleoli surrounded by fibrous tissue. Keratinization, plasmodesmata, and glandular construction were absent (**a**). High-power image of DMM (**b**). IHC demonstrated positivity for calretinin (**c**) and CAM5.2 (**d**)

A 74-year-old man was referred to our hospital with cough and dyspnea on exertion. CT revealed a large pleural effusion. Cytology class III showed bloody effusion, and MPM was suspected (Fig. 1-a, c, e). A VATS biopsy revealed proliferated spindle cells and irregular, dense, hyalinized collagen fibers (Fig. 2-a, b). The collagen fibers in the pleura were aligned irregularly, demonstrating a partial storiform pattern. IHC revealed tumor cells positive for calretinin, p53, WT1, smooth muscle actin, and desmin but negative for CD5/6 and EMA (Fig. 2-c, d). He was diagnosed with stage III DMM. Systemic chemotherapy with cisplatin and pemetrexed combination therapy was initiated but discontinued after two cycles because of renal impairment. Disease progression occurred with a 28 % increase in tumor size (Fig. 1-b, d, f), and he died of acute renal dysfunction 4.7 months after initial diagnosis.

### Case 4

A 63-year-old man was admitted to the hospital suffering from right anterior chest pain. He had pleural thickening and suspected MPM. He underwent CT-guided biopsy, but no definitive diagnosis was made. Therefore, he underwent VATS biopsy, which showed dense collagen fibers in the chest muscle and fatty tissue. The collagen fibers were mixed or fused with hyaline. Spindle cells were increased among the collagen fibers, and focal inflammatory cell invasion was noted. IHC was positive for CAM5.2 and calretinin and negative for CD5/6, AE1/AE3, HBME-1, and thrombomodulin. He was diagnosed with stage IV DMM. He received one cycle of cisplatin and gemcitabine combination chemotherapy to no effect, with rapid clinical disease progression. He died 0.9 months after the hospital admission, and autopsy confirmed DMM.

## Discussion

DPM is a relatively rare histological subtype of MPM with sarcomatoid histology, with a generally poor prognosis. We report four patients with DMM who demonstrated poor prognoses and in whom diagnosis was delayed.

The clinical behavior of MPM is characterized by local spread, large pleural effusions, and metastases to regional lymph nodes, while the sarcomatous subtype of MPM is more frequently associated with distant metastases, but with little or no effusion, and mixed mesotheliomas have intermediate features. Distant metastases occur in 60.1 % of patients with DMM [10], and a higher incidence of metastases has been reported for DMM compared with other series of MPM [5]. Among the biphasic and sarcomatous subtypes of tumors, more than 50 % of the tumor consists of dense, hypocellular collagenous tissue, and these tumors have been termed DMM. Although MPM is clearly associated with exposure to asbestos, the association between DMM and asbestos exposure is controversial [11, 12]. However, all four of the current patients had been exposed to asbestos.

Sarcomatoid components, large cellular vacuolization, infrequent psammoma bodies, and the existence of hyaluronic acid are common pathological findings [13]. It is essential to differentiate DMM from other benign lesions, such as fibrous pleurisy and solitary fibrous tumors, because of important differences in treatment and prognosis; however, it is often difficult to distinguish between these diseases both radiologically and clinically. Pathological diagnosis is the most reliable diagnostic method, although it is difficult to perform with a small amount of specimen [14]. IHC staining plays a crucial diagnostic role, in that solitary fibrous tumors are positive for CD34 and bcl-2, but negative for calretinin and HBME-1. Furthermore, collagen necrosis may provide a diagnostic clue in nearly 75 % of patients [10, 15, 16]. Given the difficulty in differentiating between DMM and fibrous pleurisy, early invasive interventions, including VATS, may be indicated if MPM is suspected. IHC staining can provide additional diagnostic information to the characteristic histological findings [11, 17]. In the current series, it was necessary to differentiate between DMM and fibrous pleurisy in Cases 1 and 4, although all cases were ultimately diagnosed definitively by histology. Recently, the classification system for DMM has been updated. Previously considered an independent histological classification in 2004, DMM is now sub-categorized under sarcomatoid histology (however, still now independently described). In addition, the role of IHC has been emphasized, and broad-spectrum staining for cytokeratins is crucial for the correct diagnosis of DMM. In addition, the criteria for distinguishing malignant mesothelioma from reactive mesothelial proliferations have been further refined. In addition, the use of p16 FISH promises to yield observations in mesothelioma pathology useful for distinguishing it from benign pleuritic [18]. Furthermore, based on a previous case report [19], positron emission tomography (PET)/CT has been clinically useful for determining a CT-guided biopsy site; however, the present cases did not undergo PET/CT.

DMM is associated with more distant metastases than other histologies [10]. The median survival time of sarcomatoid-type MPM is 5.5 months. DMM also has a poor prognosis, but the rarity of this subtype means that its detailed characteristics are poorly known. Our series included patients with advanced-stage disease, including two with stage III and two with stage IV, according to iMig staging.

The current standard of care for MPM involves multi-modality treatment. However, having difficulties with early diagnosis means that most patients are diagnosed with advanced-stage disease. MPM is characterized as highly malignant, aggressive, and refractory to local treatment, resulting in survival times of 12–36 months for localized disease and 8–14 months for advanced disease [3]. Current therapies are expected to prolong survival time and improve quality of life in patients with MPM [20]. However, the survival time of untreated patients with MPM was 11.5 months, whereas that for patients with sarcomatoid type MPM treated with supportive care was only 5–6 months [10, 11]. Furthermore, patients may take up to 1–2 years to develop symptoms [10, 21].

There is currently no definitive treatment for DMM, and it is generally managed according to MPM guidelines. National Comprehensive Cancer Network guidelines recommend chemotherapy alone [3], and the present regimen for first-line chemotherapy consists of cisplatin plus pemetrexed, based on a median overall survival of 13.3 months, compared with 12.7 months for cisplatin alone [22]. A recent phase III clinical trial demonstrated survival of 18.8 months with bevacizumab, cisplatin, and pemetrexed, compared with 16.1 months for cisplatin and pemetrexed (hazard ratio (HR) = 0.77; 95 % confidence interval (CI): 0.62–0.95; *p* = 0.0167) [23]. According to subgroup analyses, bevacizumab was associated with advantages in sarcomatoid or mixed histology MPMs (HR = 0.64; 95 % CI: 0.40–1.02 and HR = 0.82; 95 % CI: 0.64–1.06; *p* = 0.29, respectively) [23]. From this standpoint, patients diagnosed with sarcomatoid histology, including DMM, may be well-advised to add bevacizumab to their chemotherapy regimen. Carboplatin-based chemotherapy in combination with pemetrexed [24, 25] or gemcitabine are allowed [26, 27]. All of the patients in the current study were diagnosed in advanced stages. One patient underwent surgery, and the other three were treated with cisplatin-based chemotherapy, though chemotherapy was ineffective. The survival times ranged from 0.9 to 11.5 months, which indicated a poorer prognosis than for other histological subtypes of MPM, which had a median survival time of 10.5 months.

## Conclusions

We reported on four patients with DMM in whom diagnosis was only made at an advanced stage, and who had poor prognoses, as noted in previous reports.

## Additional files

Additional file 1: Figure-CT-case-1. Chest computed tomography of DMM lesions of Case 1. **Figure-path-case-1** (a) Low-power image of case 1. (b) High-power image of case 1. (ZIP 10146 kb) Additional file 2: Figure-CT-case-2. Chest computed tomography of DMM lesions of Case 2. **Figure-path-case-2** (a) Low-power image showed focal proliferation of cuboidal atypical cells with round nuclei and prominent nucleoli surrounded by fibrous tissue. Keratinization, plasmodesmata, and glandular construction were absent. (b) High-power image of DMM. IHC demonstrated positivity for (c) calretinin and (d) CAM5.2. (ZIP 11113 kb) Additional file 3: Figure-CT-case-3. Chest computed tomography of DMM lesions of Case 3. **Figure-path-case-3** Pathological findings of VATS-resected specimen of Case 3. (a) Low-power image showed focal proliferation of cuboidal atypical cells with round nuclei and prominent nucleoli surrounded by fibrous tissue. Keratinization, plasmodesmata, and glandular construction were absent. (b) High-power image of DMM. IHC demonstrated positivity for (c) calretinin and (d) CAM5.2. (ZIP 10747 kb) Additional file 4: Figure-CT-case-4. Chest computed tomography of DMM lesions of Case 4. **Figure-path-case-4** (a) Low-power image of case 4. (b) High-power image of case 4. (ZIP 7796 kb)

## Abbreviations

DMM: Desmoplastic malignant mesothelioma
IHC: Immunohistochemistry
MPM: Malignant pleural mesothelioma
