# Supplementary figures and images for: Malignant mesothelioma of the pleura with desmoplastic histology: a case series and literature review
Source: BMC Cancer. 2016 Sep 6;16(1):718. doi: 10.1186/s12885-016-2745-8 (PMC5012075; doi:10.1186/s12885-016-2745-8)

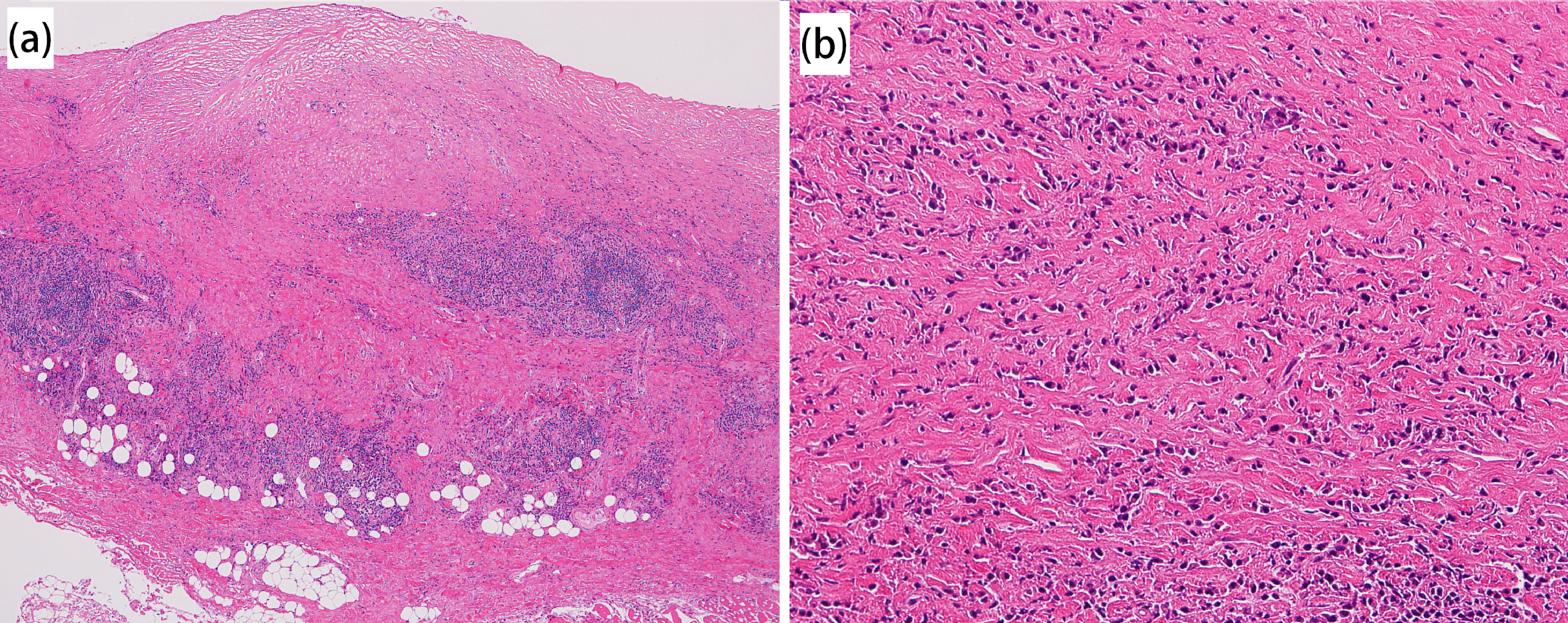

Supplement: Additional file 1: Figure-CT-case-1. — Chest computed tomography of DMM lesions of Case 1. Figure-path-case-1 (a) Low-power image of case 1. (b) High-power image of case 1. (ZIP 10146 kb) [file 12885_2016_2745_MOESM1_ESM.zip › Supplementary figure 1/1-A-BR3.jpg]

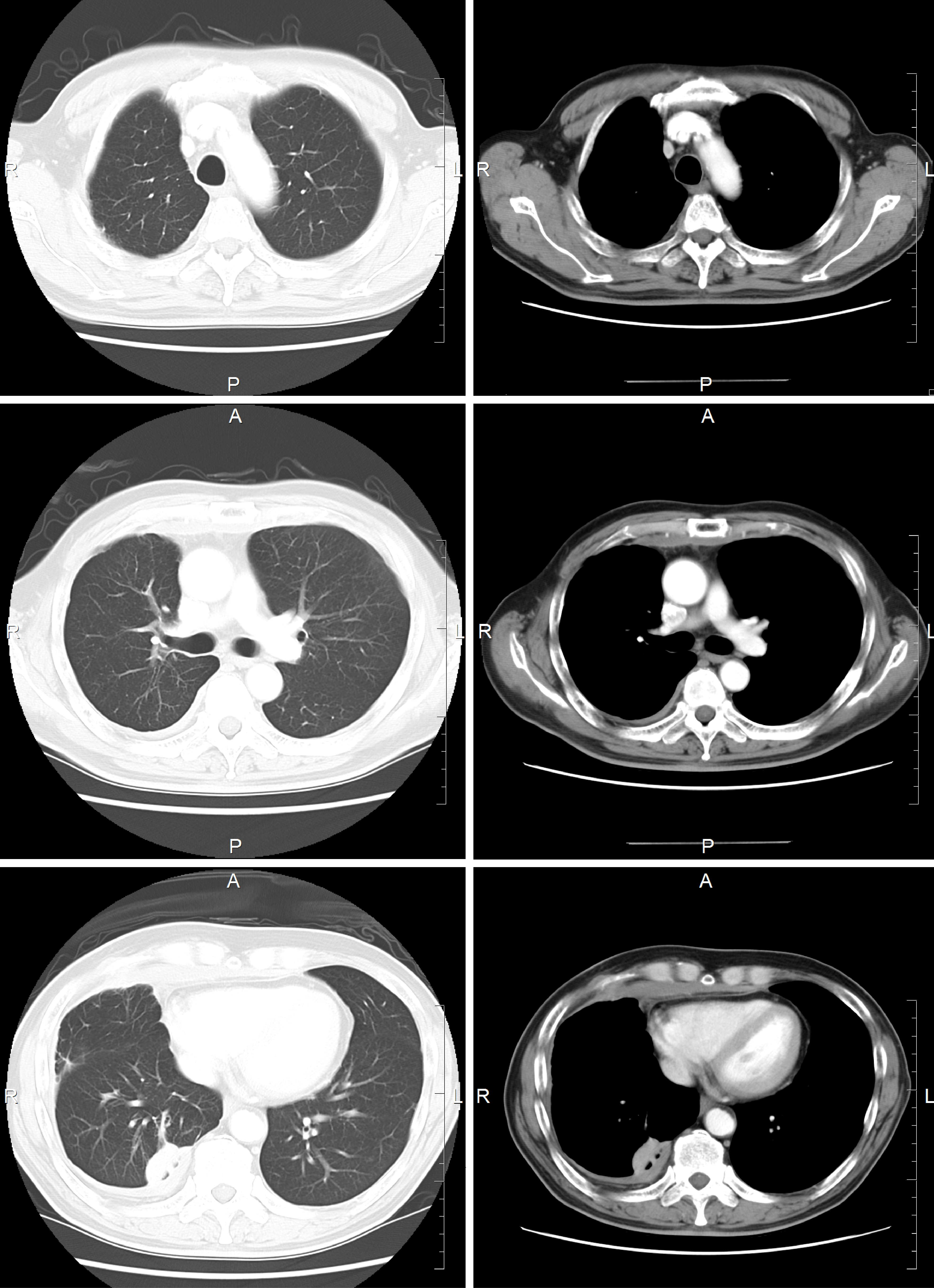

Supplement: Additional file 1: Figure-CT-case-1. — Chest computed tomography of DMM lesions of Case 1. Figure-path-case-1 (a) Low-power image of case 1. (b) High-power image of case 1. (ZIP 10146 kb) [file 12885_2016_2745_MOESM1_ESM.zip › Supplementary figure 1/Case-1p-CTR3.jpg]

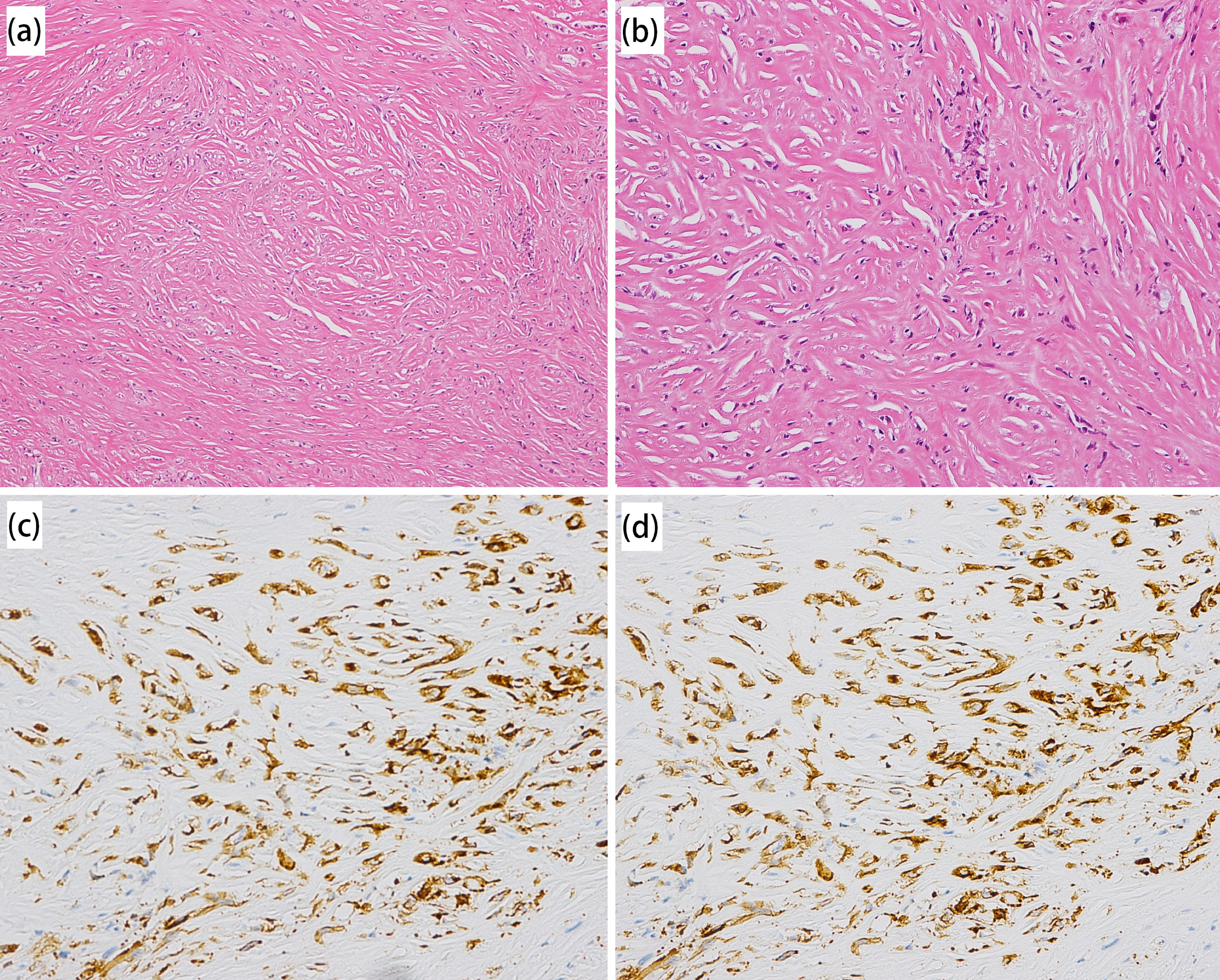

Supplement: Additional file 2: Figure-CT-case-2. — Chest computed tomography of DMM lesions of Case 2. Figure-path-case-2 (a) Low-power image showed focal proliferation of cuboidal atypical cells with round nuclei and prominent nucleoli surrounded by fibrous tissue. Keratinization, plasmodesmata, and glandular construction were absent. (b) High-power image of DMM. IHC demonstrated positivity for (c) calretinin and (d) CAM5.2. (ZIP 11113 kb) [file 12885_2016_2745_MOESM2_ESM.zip › Supplementary figure 2/2-A-DR3.jpg]

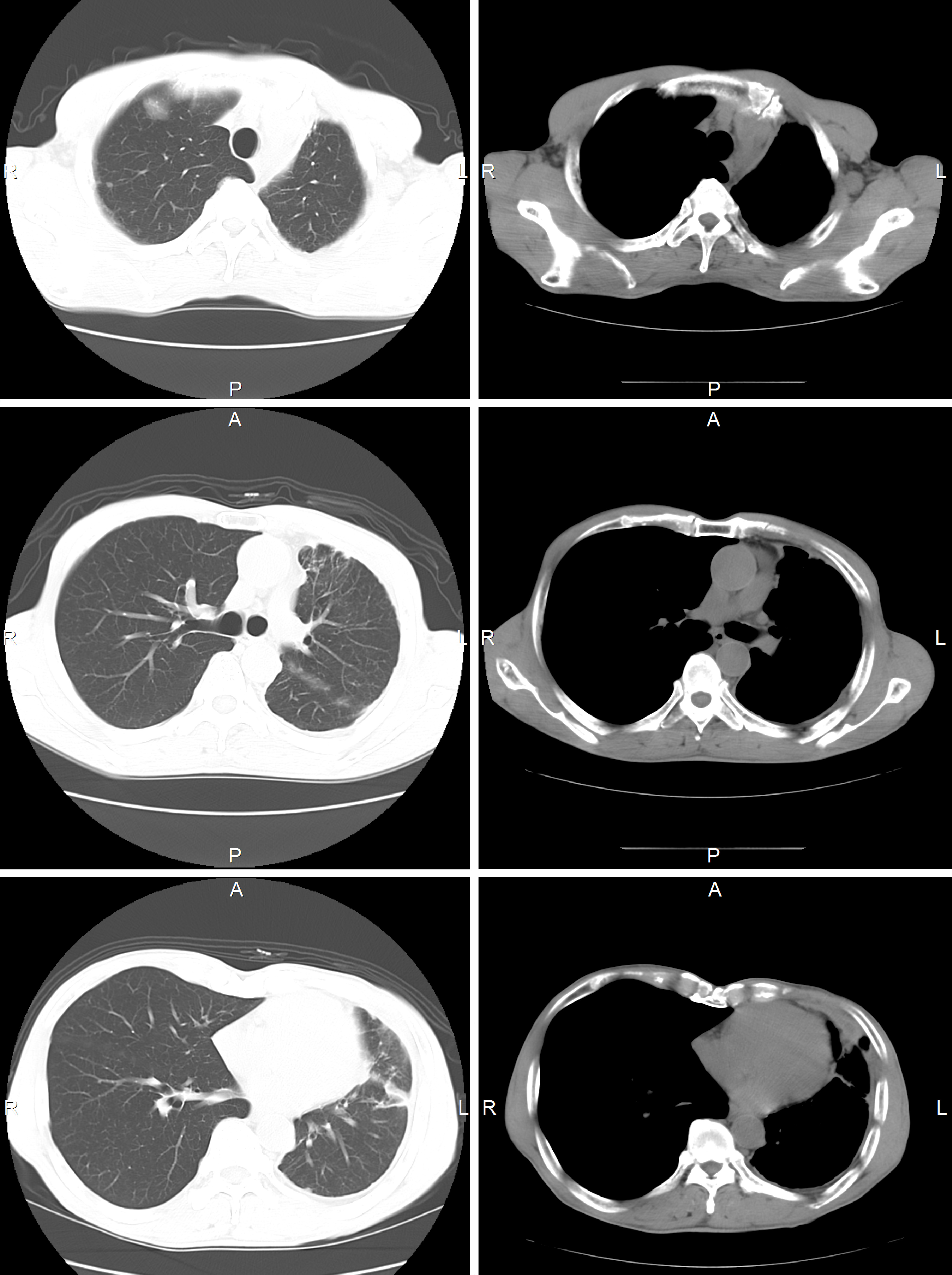

Supplement: Additional file 2: Figure-CT-case-2. — Chest computed tomography of DMM lesions of Case 2. Figure-path-case-2 (a) Low-power image showed focal proliferation of cuboidal atypical cells with round nuclei and prominent nucleoli surrounded by fibrous tissue. Keratinization, plasmodesmata, and glandular construction were absent. (b) High-power image of DMM. IHC demonstrated positivity for (c) calretinin and (d) CAM5.2. (ZIP 11113 kb) [file 12885_2016_2745_MOESM2_ESM.zip › Supplementary figure 2/Case-2q-CTR3.jpg]

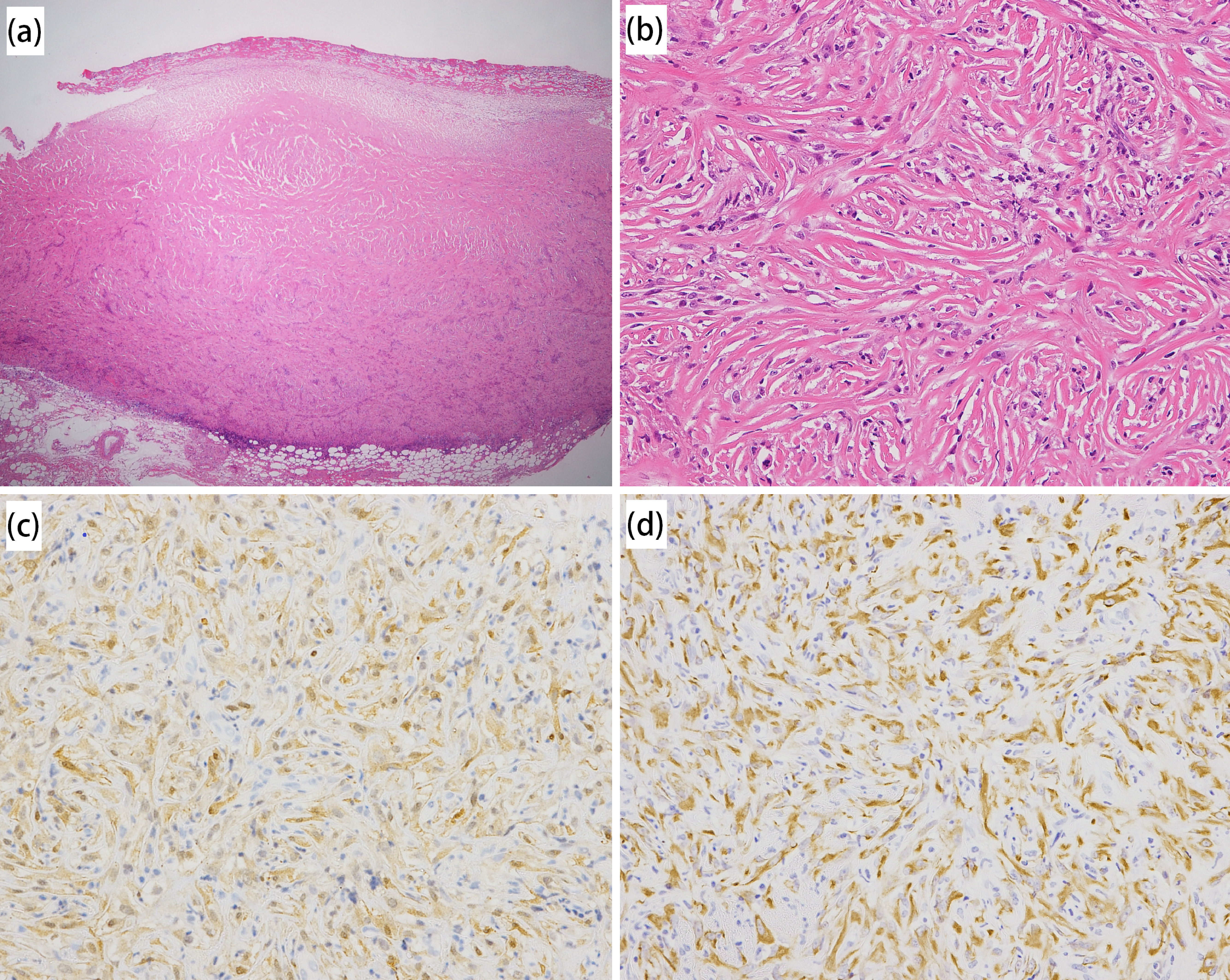

Supplement: Additional file 3: Figure-CT-case-3. — Chest computed tomography of DMM lesions of Case 3. Figure-path-case-3 Pathological findings of VATS-resected specimen of Case 3. (a) Low-power image showed focal proliferation of cuboidal atypical cells with round nuclei and prominent nucleoli surrounded by fibrous tissue. Keratinization, plasmodesmata, and glandular construction were absent. (b) High-power image of DMM. IHC demonstrated positivity for (c) calretinin and (d) CAM5.2. (ZIP 10747 kb) [file 12885_2016_2745_MOESM3_ESM.zip › Supplementary figure 3/3-A-DR3.jpg]

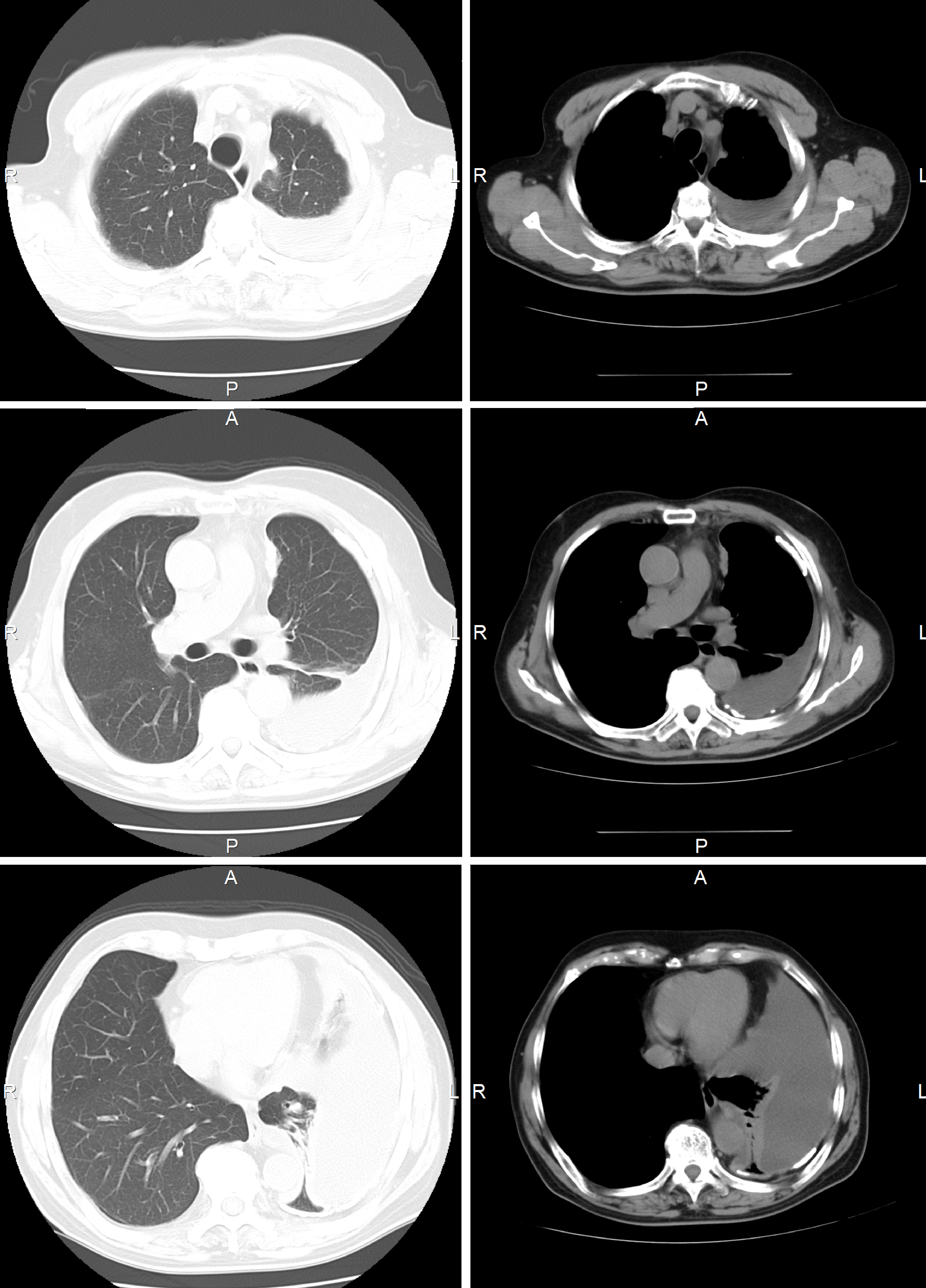

Supplement: Additional file 3: Figure-CT-case-3. — Chest computed tomography of DMM lesions of Case 3. Figure-path-case-3 Pathological findings of VATS-resected specimen of Case 3. (a) Low-power image showed focal proliferation of cuboidal atypical cells with round nuclei and prominent nucleoli surrounded by fibrous tissue. Keratinization, plasmodesmata, and glandular construction were absent. (b) High-power image of DMM. IHC demonstrated positivity for (c) calretinin and (d) CAM5.2. (ZIP 10747 kb) [file 12885_2016_2745_MOESM3_ESM.zip › Supplementary figure 3/Case-3r-CTR3.jpg]

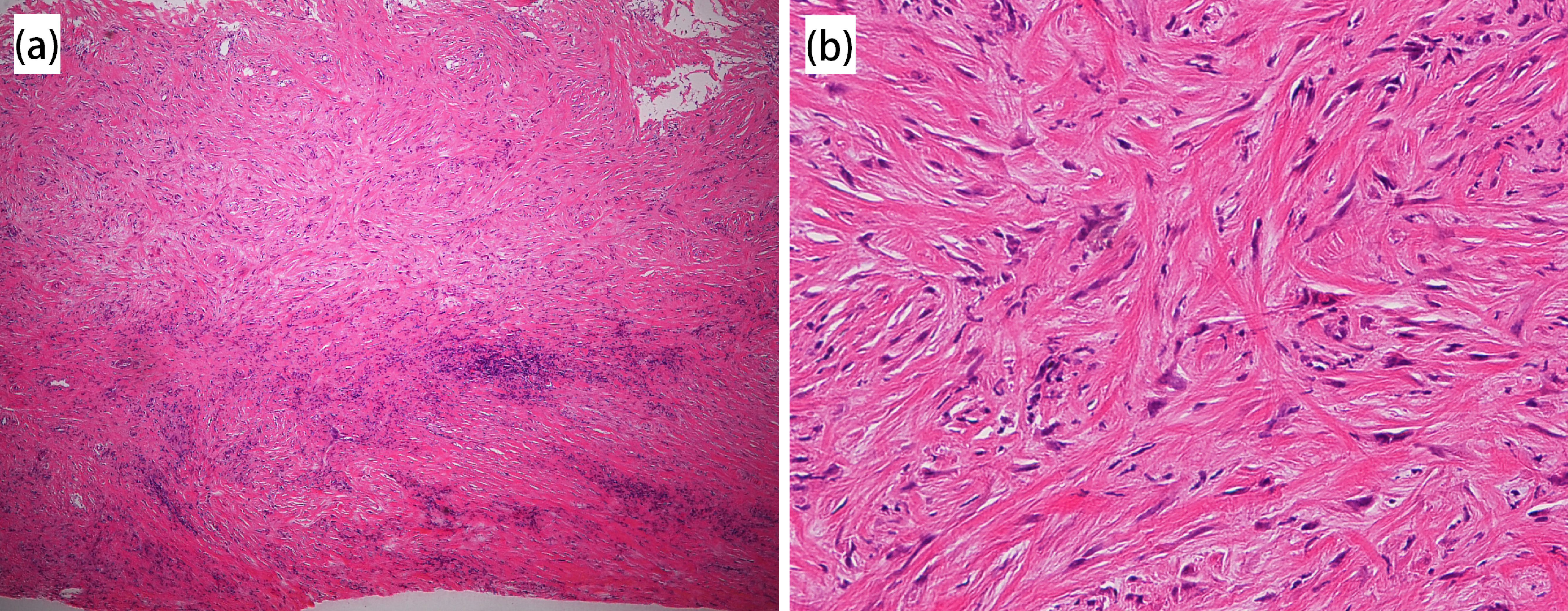

Supplement: Additional file 4: Figure-CT-case-4. — Chest computed tomography of DMM lesions of Case 4. Figure-path-case-4 (a) Low-power image of case 4. (b) High-power image of case 4. (ZIP 7796 kb) [file 12885_2016_2745_MOESM4_ESM.zip › Supplementary figure 4/4-A-BR3.jpg]

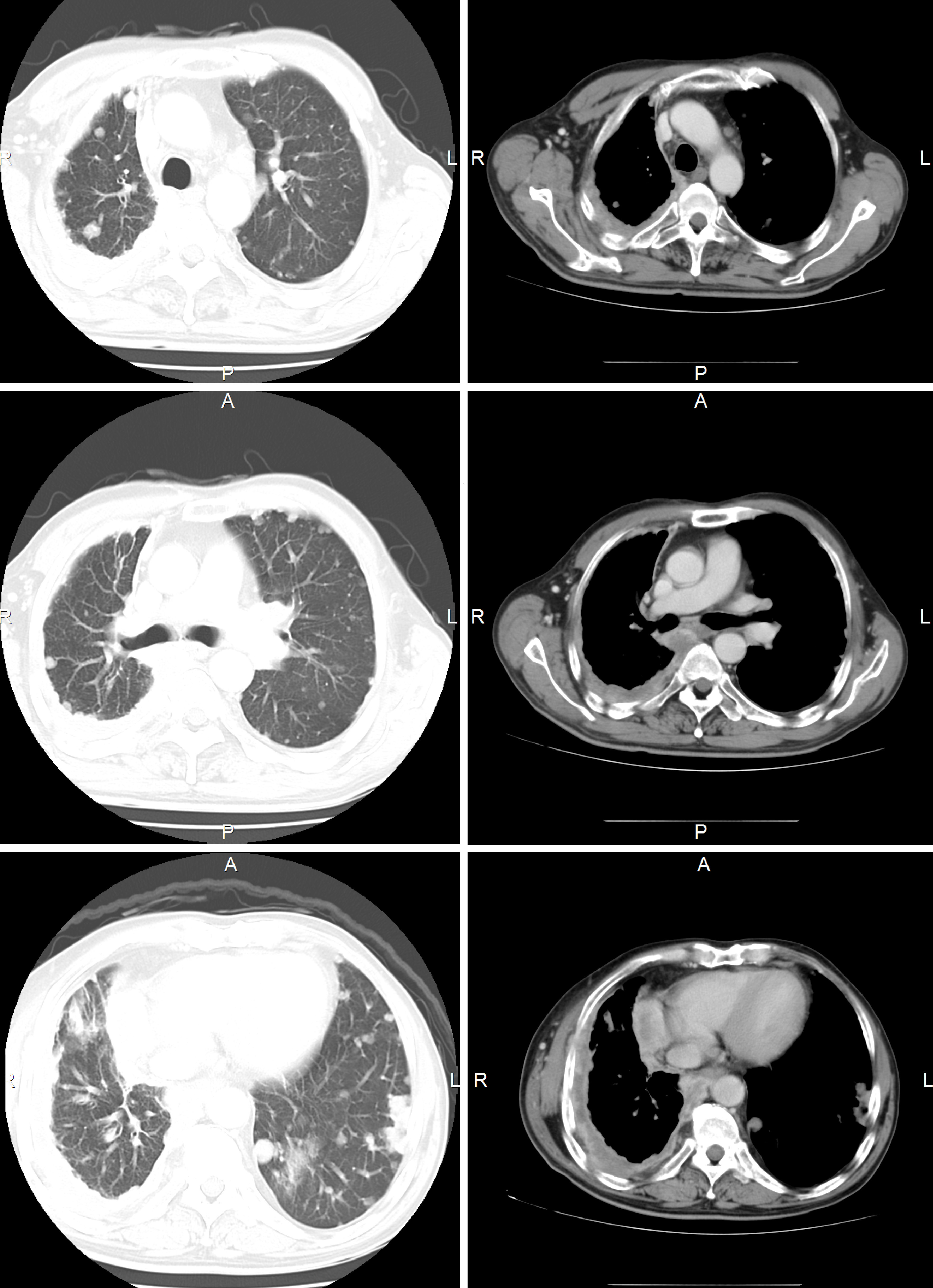

Supplement: Additional file 4: Figure-CT-case-4. — Chest computed tomography of DMM lesions of Case 4. Figure-path-case-4 (a) Low-power image of case 4. (b) High-power image of case 4. (ZIP 7796 kb) [file 12885_2016_2745_MOESM4_ESM.zip › Supplementary figure 4/Case-4s-CTR3.jpg]
